# Effect of tissue flossing on eccentric exercise-induced muscle damage: A randomized controlled trial

**DOI:** 10.5114/biolsport.2025.147019

**Published:** 2025-02-05

**Authors:** Jianxin Chen, Fang Cheng, Jinqi Yang, Jinfeng Yang, Jianxin Ran, Yuanpeng Liao

**Affiliations:** 1 Institute of Sports Medicine and Health, Chengdu Sport University, Chengdu 610041, China; 2 Affiliated Hospital of Chengdu Sport University, Chengdu Sport University, Chengdu 610041, China

**Keywords:** Creatine kinase Fascia, Delayed onset muscle soreness, Tissue flossing, Muscle strength

## Abstract

Tissue flossing (TF) is a novel technique designed to improve muscle and joint function and performance. This study investigated the effects of TF on exercise-induced muscle damage (EIMD) in 24 males randomized into TF (n = 12) or control (CON, n = 12) groups. Participants performed dominant thigh quadriceps isokinetic eccentric exercises to induce EIMD, 10 sets × 12 repetitions. The TF group performed TF interventions (active movement during floss band wrapping, three times a session) immediately after and 24–48 h postexercise. Creatine kinase (CK), knee extensor eccentric strength, pressure pain threshold (PPT), and deep fascia sliding (DFS) of quadriceps were measured at baseline and 24, 48, and 72 h postexercise. CK levels significantly increased over time postexercise, with CON showing a greater increase than TF (all p < 0.05). Knee extensor eccentric strength returned to baseline levels for TF at 48 h (= 0.081) and CON at 72 h (= 0.058), with TF showing greater improvement postexercise (all p < 0.05). Both groups’ rectus femoris PPT returned to baseline at 72 h (TF, = 0.303; CON, = 0.272). However, only TF returned to baseline in the vastus medialis PPT and DFS at 72 h and the vastus lateralis DFS at 48 h (all p > 0.05). Furthermore, the PPT and DFS of the vastus medialis and lateralis were significantly greater in TF than in CON over time postexercise (all p < 0.05). Overall, TF is an effective strategy for alleviating EIMD after high-intensity exercise.

## INTRODUCTION

Exercise-induced muscle damage (EIMD) mainly results from unaccustomed physical activity, particularly when it involves numerous eccentric contractions [1]. This damage process typically leads to delayed onset muscle soreness (DOMS), which generally begins a few hours postexercise and gradually peaks within several days [2], along with a temporary reduction in muscle strength [3]. Many theories have been advanced to explain the damage process in recent decades [4], but the exact mechanism is still inconclusive. In recent years, following extensive fascia research, the fascia was believed to be a major source of the pain after eccentric exercise [5, 6]. Furthermore, dysfunction of deep fascia sliding has been observed during the period of DOMS, which correlates with the subsequent improvement of DOMS [7]. Thus, the function of fascia sliding may have an important influence on DOMS; however, there are still no comprehensive studies investigating this relationship.

EIMD has been hypothesized as an adaptation process in muscular hypertrophy [8]. However, it can result in DOMS and a decrease in muscle function within a few days to a couple of weeks following exercise, affecting sport performance for both recreational individuals and athletes [9]. Particularly during the competitive phase of a sports season, minimizing performance impairments is crucial, as even minor decreases in performance can significantly impact outcomes. Therefore, it is essential to prescribe effective recovery techniques to reduce impairment from EIMD and accelerate recovery after exercise. Currently, several interventions have been suggested to alleviate EIMD and DOMS, including cold water immersion, electrical stimulation, and vibration [10, 11]. However, these interventions are too costly for the majority of people and, in some cases, have been considered ineffective [12].

Tissue flossing (TF) is a novel technique designed to help athletes return to competition sooner, prevent injuries, lessen pain, and increase range of motion (RoM). It has been proven that the RoM of the associated joint can be enhanced by the application of TF to the soft tissue or the joint [13]. However, the effectiveness of TF in recovering from EIMD remains uncertain, with a limited number of studies yielding inconsistent results [14, 15]. These inconsistencies may arise from methodological variations in comparison to current studies on TF [13, 16]. According to “fascia shearing” proposed by Starrett and Cordoza, it is recommended that individuals strive to accomplish the maximum RoM of the treatment site during wrapping rather than only passively wrapping [17]. This theory suggests the combination of TF tight compression and muscle contraction during active movement could generate mechanical stress and heat, facilitating a transition of the fascia from a solid gel to a fluid-like state, thereby increasing fascia stretching and mobility [18]. However, there is still a dearth of evidence-based support for this theory.

Therefore, the main purpose of this study was to determine whether TF has positive effects on EIMD and DOMS. Secondly, the study aimed to evaluate whether TF could accelerate recovery postexercise by improving deep fascia sliding.

## MATERIALS AND METHODS

### Participants

The study included 24 healthy, physically inactive males. G*Power (version 3.1.9.7, Heinrich Heine University Düsseldorf, Germany) determined the sample size. The evaluated design parameters were: α = 0.05; (1-β) = 0.8; f = 0.25; F test; repeated measures ANOVA; 2 groups; 4 measurements. A sample size of 24 (12 in each group) was calculated. The inclusion criteria were: 1) age 18–25 years; 2) screened by the Physical Activity Readiness Questionnaire without any known cardiovascular or musculoskeletal disease or injury; and 3) no physical activity performed in the past 6 months. The exclusion criteria included: 1) taking medication; 2) Latex allergy; 3) smoking or alcohol consumption during the period of the study. All participants were right-foot dominant and randomly assigned to a TF intervention (TF, n = 12) or control group (CON, n = 12). All participants were formally enrolled in the study after voluntarily signing the Subject Informed Consent Form. The study was approved by the Chengdu Sport University Ethical Committee (approval No. 50-2023) and followed the Declaration of Helsinki.

### Experimental design and procedure

**FIG. 1:**
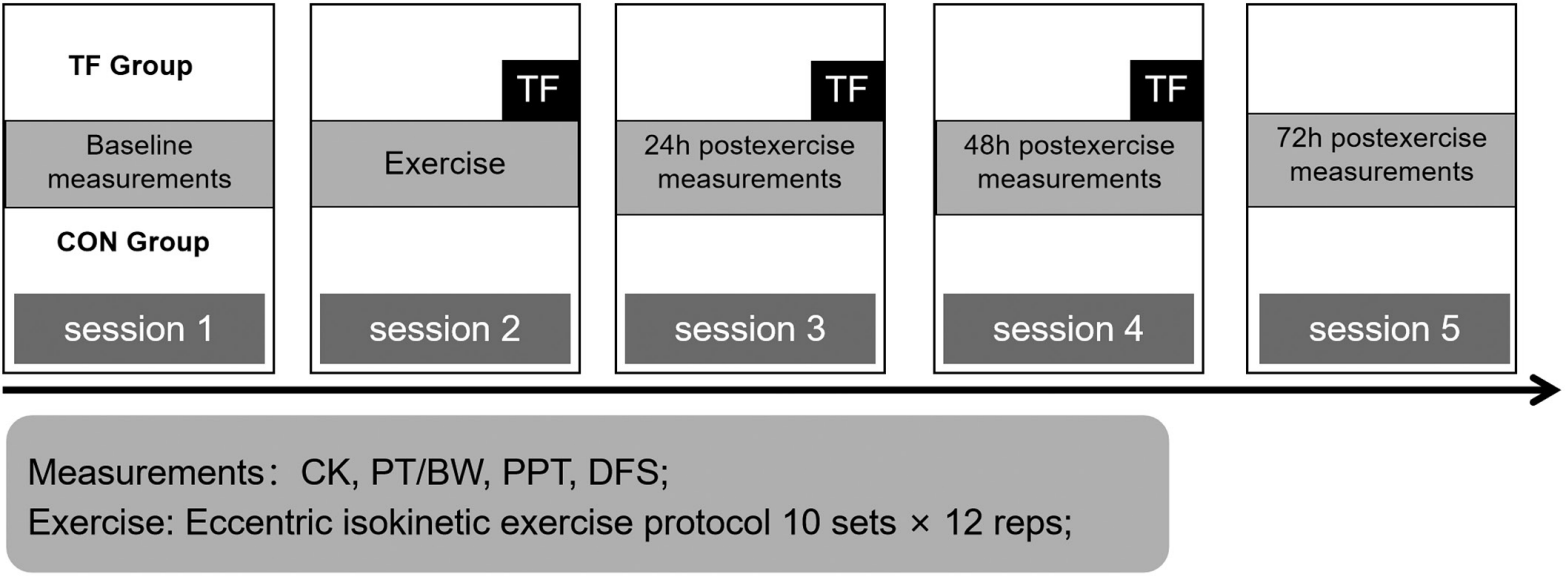
Experimental protocol of the study. The tissue flossing group (TF) received TF intervention at session 2, 3, and 4, while the control group (CON) received no intervention. CK, creatine kinase; PT/BW, peak torque / body weight; PPT, pressure pain threshold; DFS, deep fascia sliding.

Intervention (TF vs. CON) and time (baseline, 24, 48, and 72 h postexercise) were the between- and within-group factors in a parallel-group design. Each subject participated in five laboratory sessions (Figure 1). In the first session, baseline measures and participant characteristics were recorded. An InBody 570 body composition tester (InBody Ltd, Seoul, Korea) evaluated body fat, and a HK-6000-ST (Hengkonka Ltd, Shenzhen, China) height and weight tester measured height and weight. All participants performed the same quadriceps eccentric exercises during the second session. Only the TF group underwent TF immediately postexercise and again at 24 and 48 h postexercise (sessions 2–4). The CON group performed the same exercises as the TF group but did not receive any additional intervention. Creatine kinase (CK) activity, knee extensor eccentric strength, pressure pain threshold, and deep fascia sliding of the quadriceps were measured at baseline, 24, 48, and 72 h postexercise (sessions 1 and 3–5).

### Eccentric exercise

Knee eccentric flexion exercises with an isokinetic dynamometer (CON-TREX-MJ, PHYSIOMED, Germany) caused quadriceps muscle injury and DOMS in the dominant lower extremity [19]. The exercise intensity was as follows: 10 sets of 12 repetitions, for a total of 120 times, with an inter-group interval of 30 seconds. Two sets of adaptive exercises were conducted before the formal training began. The active RoM was 30°–90° knee flexion and 60°/s angular velocity. Participants sat on the dynamometer and only did eccentric exercises.

### Intervention

The TF group received a floss band (Flossband Plum, 3.5 m length, 5 cm width, Sanctband, Malaysia) wrapped around the dominant thigh from above the superior patella pole to the femur greater trochanter, with a 50% overlap [13]. After 20 active squats, the floss band was removed. This method took 2 minutes, a 1-minute break, and was performed three times per session, for a total of 60 squats. A total of three intervention sessions were conducted separately: immediately postexercise, and at 24 and 48 h postexercise. All the TF interventions were administered by the same researcher.

The skin-floss band interface pressure was measured to determine compression (mmHg) using the kikuhime pressure monitor (MediGroup, Melbourne, Australia), a valid (ICC = 0.99, CV = 1.1%) and reliable (CV = 4.9%) sports equipment [20]. The monitor probe was midway between the superior patella pole and the femur greater trochanter. Previous study [13] indicate that a pressure limit of 167.3 ± 24.6 mmHg is suitable. The average pressure for the TF group in this experiment was 162 ± 8.80 mmHg.

### Creatine kinase

Venous blood (2 mL) was collected from the participant’s antecubital vein in each measurement by a qualified nurse. The serum was separated and stored at −80°C after centrifugating the sample for 10 minutes at 4°C and 3000 rpm. Serum CK activity was measured using an enzymatic kinetic assay method (BS-240, Mindray, Shenzhen, China).

### Knee extensor eccentric strength

Peak torque/body weight (PT/BW) was used to measure knee extensor eccentric strength using the isokinetic dynamometer, the same apparatus employed for eccentric exercise. The equipment was adjusted to meet the exercise protocol’s RoM and angular velocity. Prior to testing, participants did two warm-ups. The formal testing includes performing the exercise five times continuously and averaging the results.

### Press pain threshold

The vastus medial (VM-PPT), vastus lateral (VL-PPT), and rectus femoris (RF-PPT), which was halfway between the midpoint of the inguinal region and the upper pole of the patella, were measured for pressure pain threshold (PPT). A semi-permanent pen indicated and reinforced the measuring areas after each measurement. Participants were told not to wash the marks during the trial. In measurement, the researcher steadily raised pressure at the designated position using the Kikuhime gadget probe until the participant reported a painful sensation. The value was immediately reported by another researcher. For each region, three measurements were taken and the average was reported. The same researcher measured all patients’ pain.

### Deep fascia sliding

Horizontal movement distance measured anterior thigh deep fascia sliding. The measurement positions were the same as for PPT, which included the deep fascia sliding of vastus medial (VM-DFS), vastus lateral (VL-DFS), and rectus femoris (RF-DFS). Participants sat on the dynamometer like the exercise protocol. The dynamometer generated 5°/s knee passive movement and 30°–90° RoM knee flexion. This velocity did not cause reflexive muscular contraction [21]. The testing site was equipped with a high-resolution (US) device probe (Philips CX50, the Netherlands) to record passive deep fascia sliding. To avoid test posture action, participants were instructed to perform 10 adaption practices and rest before each formal measurement. The probe was parallel to the muscle and perpendicular to the skin to avoid extrusion when measuring. Each video began at 30° knee flexion and ended at 90°. The video was rerecorded if the deep fascia motion artefact arose during the test.

The acquisition movies were examined using MATLAB’s frame-by-frame cross-correlation technique. The widely used Dilley et al. approach [22] quantifies tissue displacement with great reliability (ICC: 0.77–0.99) [23]. The mean horizontal displacement of five equidistant zones of interest in each testing position was determined in the video.

### Statistics

All data was analyzed using SPSS 22.0 (IBM Corp., Armonk, NY, USA). Results are presented as mean ± standard deviation (mean ± SD). The Shapiro-Wilk test assessed distribution. A two-way repeated measures ANOVA was used to determine group-time interactions for normal distribution variables. The Greenhouse-Geisser adjustment value was used when variables failed Mauchly’s sphericity test. A simple main effect analysis utilizing Bonferroni, was conducted following an ANOVA that indicated an interaction, along with multiple comparisons within-group factor. An analysis of the main effects of groups and times was conducted using Bonferroni when no interaction was present, to ascertain the separate effects of the between-group factor (groups) or the within-group factor (times) on the outcome. Nonparametric tests (independent and related samples) were used to analyze nonnormal variables. In this study, PT/BW, VM-PPT, VL-PPT, VM-DFS, VL-DFS, and RF-DFS had normal distributions, but CK and RF-PPT did not. Additionally, the partial eta squared (ƞ^2^_p_) was used to determine ANOVA variable effect sizes. Differences of 0.01, 0.06, and 0.14 are modest, medium, and big. G*Power calculated the effect sizes of variables investigated by non-parametric tests, with values of 0.2, 0.5, and 0.8 reflecting small, medium, and large differences.

## RESULTS

Table 1 lists all participants’ physical traits. No significant differences were found across groups (*p* > 0.05). CK activity, knee extensor eccentric strength, PPT, and deep fascia sliding were not significantly different between CON and TF groups at baseline (*p* > 0.05).

**TABLE 1:** Physical characteristics for participants.

	CON (n = 12)	TF (n = 12)
Age (years)	21.00 ± 2.04	21.08 ± 1.78
Height (cm)	175.91 ± 6.48	176.75 ± 4.63
Weight (kg)	72.41 ± 5.50	73.58 ± 3.17
Rate of body fat (%)	19.16 ± 3.01	18.08 ± 3.89

Note: CON, control group; TF, group that received tissue flossing intervention.

**FIG. 2:**
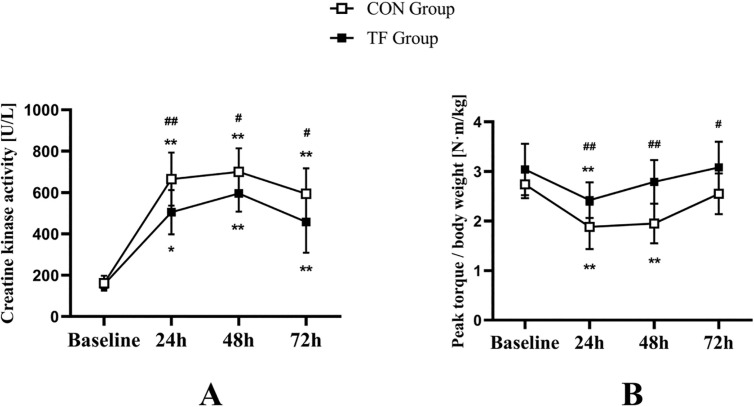
The tissue flossing (TF) group and the control (CON) group showed results for creatine kinase (CK) activity (A) and knee extensor eccentric strength (B) at baseline, 24 h, 48 h, and 72 h postexercise. *p < 0.05, **p < 0.01 compared to baseline; #p < 0.05, ##p < 0.01 significant between groups difference.

Both groups had maximal CK activity at 48 h postexercise but did not revert to baseline at any time period: TF at 72 h (*p* = 0.002, d = 2.83), CON at 72 h (*p* = 0.002, d = 4.73). CK increase over time postexercise was larger in the CON group than in the TF group (at 24 h, *p* = 0.007, d = 1.350; at 48 h, *p* = 0.045, d = 1.023; at 72 h, *p* = 0.028, d = 0.991) (Figure 2).

Both groups’ PT/BW reached their lowest value 24 h postexercise and recovered gradually. PT/BW revealed significant time-group interaction (F = 6.003, *p* = 0.010, ƞ^2^_p_ = 0.214), with substantial simple group effects at 24 h (F = 10.359, *p* = 0.004, ƞ^2^_p_ = 0.320), 48 h (F = 23.452, *p* < 0.001, ƞ^2^_p_ = 0.516), and 72 h (F = 7.521, *p* = 0.012, ƞ^2^_p_ = 0.255). A substantial simple time impact was found in both the TF (F = 35.61, *p* < 0.001, ƞ^2^_p_ = 0.842) and CON (F = 22.315, *p* < 0.001, ƞ^2^_p_ = 0.770) groups. PT/BW recovered to baseline for the TF group at 48 h (*p* = 0.081) and the CON group at 72 h (*p* = 0.058) (Figure 2).

**FIG. 3:**
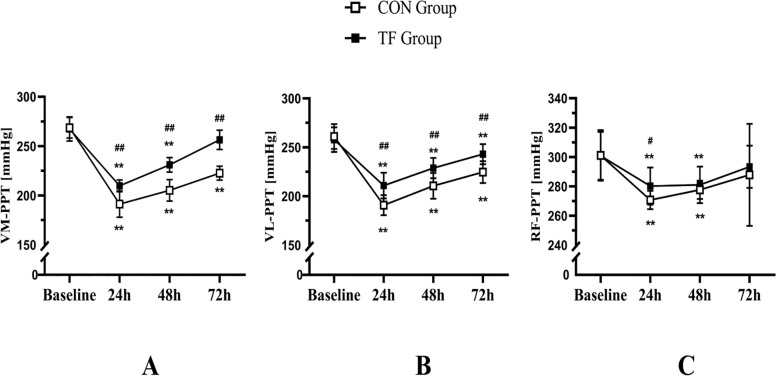
The tissue flossing (TF) group and the control (CON) group showed results for pressure pain threshold of the vastus medial (VM-PPT, A), vastus lateral (VL-PPT, B), and rectus femoris (RF-PPT, C) at baseline, 24 h, 48 h, and 72 h postexercise. *p < 0.05, **p < 0.01 compared to baseline; #p < 0.05, ##p < 0.01 significant between groups difference.

VM-PPT showed a significant time × group interaction (F = 15.790, *p* < 0.001, ƞ^2^_p_ = 0.418), as well as VL-PPT (F = 5.267, *p* = 0.003, ƞ^2^_p_ = 0.193), with a significant simple group effect at 24 h (VM-PPT, F = 19.156, *p* < 0.001, ƞ^2^_p_ 2 p = 0.456; VL-PPT, F = 17.454, *p* < 0.001, ƞ^2^_p_ = 0.442), 48 h (VM-PPT, F = 45.997, *p* < 0.001, ƞ^2^_p_ = 0.676; VL-PPT, F = 13.886, *p* < 0.001, ƞ^2^_p_ = 0.387), and 72 h (VM-PPT, F = 94.827, *p* < 0.001, ƞ^2^_p_ = 0.812; VL-PPT, F = 17.404, *p* < 0.001, ƞ^2^_p_ = 0.442). Both the TF group (VM-PPT, F = 84.946, *p* < 0.001, ƞ^2^_p_ = 0.927; VL-PPT, F = 36.639, *p* < 0.001, ƞ^2^_p_ = 0.846) and the CON group (VM-PPT, F = 112.239, *p* < 0.001, ƞ^2^_p_ = 0.944; VL-PPT, F = 59.315, *p* < 0.001, ƞ^2^_p_ 0.899) exhibited significant simple time effects. After 72 hours postexercise, only the TF group restored to baseline VM-PPT values (*p* = 0.087), but the CON group did not (*p* < 0.001). In VL-PPT, neither the TF (*p* = 0.030) nor the CON (*p* < 0.001) groups returned to baseline levels after 72 hours. RF-PPT reached its minimum value at 24 h postexercise and reverted to baseline at 72 h (*p* = 0.272 and 0.303 for the CON and TF groups, respectively). The TF group had higher RF-PPT at 24 h than the CON group (*p* = 0.029, d = 0.952) (Figure 3).

**FIG. 4:**
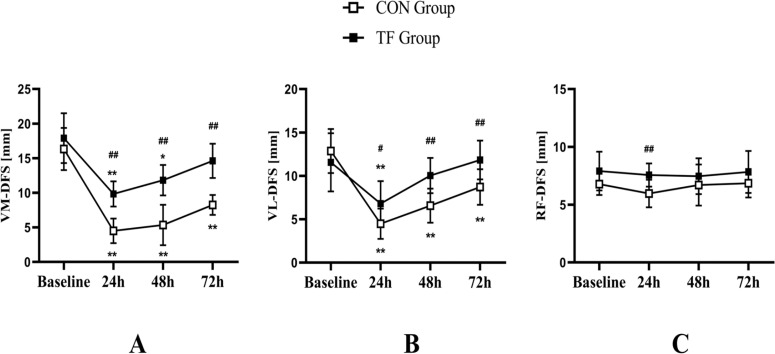
The tissue flossing (TF) group and the control (CON) group showed results for deep fascia sliding of the vastus medial (VM-DFS, A), vastus lateral (VL-DFS, B), and rectus femoris (RF-DFS, C) at baseline, 24 h, 48 h, and 72 h postexercise. *p < 0.05, **p < 0.01 compared to baseline; #p < 0.05, ##p < 0.01 significant between groups difference.

VM-DFS showed a significant time × group interaction (F = 4.739, *p* = 0.005, ƞ^2^_p_ = 0.177), as well as VL-DFS (F = 4.717, *p* = 0.005, ƞ^2^_p_ = 0.177), with a significant simple group effect at 24 h (VM-DFS, F = 51.758, *p* < 0.001, ƞ^2^_p_ = 0.702; VL-DFS, F = 6.567, *p* = 0.018, ƞ^2^_p_ = 0.230), at 48 h (VM-DFS, F = 35.935 *p* < 0.001, ƞ^2^_p_ = 0.620; VL-DFS, F = 17.849, *p* < 0.001, ƞ^2^_p_ = 0.448), and 72 h (VM-DFS, F = 58.892, *p* < 0.001, ƞ^2^_p_ = 0.728; VL-DFS, F = 12.631, *p* = 0.002, ƞ^2^_p_ = 0.365). The TF group (VM-DFS, F = 18.385, *p* < 0.001, ƞ^2^_p_ = 0.734; VL-DFS, F = 7.899, *p* = 0.001, ƞ^2^_p_ = 0.542) and the CON group (VM-DFS, F = 31.125, *p* < 0.001, ƞ^2^_p_ = 0.824; VL-DFS, F = 17.071, *p* < 0.001, ƞ^2^_p_ = 0.719) both demonstrated significant simple time effect. Only the TF group returned to baseline levels post-exercise (*p* = 0.061 and 0.776 for VM-DFS at 72 h and VL-DFS at 48 h, respectively), where- as the CON group did not at 72 h (VM-DFS, *p* < 0.001; VL-DFS, *p* = 0.002). The study found no significant interaction between time and group for RF-DFS (*p* = 0.543) or time main impact (*p* = 0.418). = However, RF-DFS showed a group main effect (F = 12.436, *p* = 0.002, ƞ^2^_p_ = 0.361) (Figure 4).

## DISCUSSION

The study investigated the effects of a novel technique, TF, on EIMD. Results indicate that TF is an effective intervention for mitigating EIMD and promoting recovery following high-intensity eccentric exercise. This study also provides a novel perspective on EIMD research by examining deep fascia sliding before and after eccentric exercise. This is the first study to examine TF’s “fascia shearing” effects. The present experiment’s decrease in fascia movement postexercise suggests that reduced deep fascia sliding may be another EIMD mechanism. The study observed a significant decline in knee extensor eccentric strength, PPT VM-DFS, and VL-DFS, followed by a progressive recovery. The TF and CON groups’ variables show that TF aids recovery. Additionally, the significant difference in CK activity between the TF and CON groups suggests that TF reduces post-exercise muscle damage. Thus, TF serves as a reusable tool with a short intervention time, making it an effective method for alleviating EIMD in exercisers.

The mechanism by which TF functions in EIMD is related to multiple factors. The effects of EIMD on athletes or recreationally active individuals are mainly DOMS and decreased muscle strength, which results in reduced exercise performance [24, 25]. The reason for DOMS remains unclear, although many theories have attempted to explain it [26]. Previously, biopsy results showed micro-ruptures in the sarcomeres following eccentric exercise, causing edema and enhanced nociceptors [27, 28]. Inflammatory cells enter wounded muscle tissue and release pain-modulating substances, which may worsen pain perception [29]. According to the gate control theory, compression treatment selectively activates large rapid-conduction A fibers, releasing inhibitory neurotransmitters and blocking nociceptive impulses from C fibers and the lateral spinothalamic tract in the spinal cord from reaching the thalamus’ pain center, relieving pain [30, 31]. By using a mechanism similar to the pressurization accessed by the Kikuhime pressure monitor, the intervention modality used in this study may also lessen DOMS following exercise. However, it is important to note that the key distinctions between TF and other pressure therapies are the application time and pressure. TF is characterized by higher intensity pressure applied for shorter durations. Moreover, the higher pressure of the TF does not necessarily mean better results. Wienke et al. [32] reported terrible pain, skin color changes, hematomas, and numbness in TF recipients, although their study did not monitor pressure, which may have caused excessive pressure. There is no unanimity on TF’s ideal pressure range. The pressure range in this trial was 162 ± 8.80 mmHg, with no adverse reactions reported. Future studies should observe the relationship between pressure and side effects through pressure monitoring equipment.

Traditional muscle tissue-based pathological models may have some limitations in explaining the delayed features of DOMS and persistent pain postexercise [33], and this experiment validated the reduction in deep fascia sliding of pertinent tissue following eccentric exercise, which could offer a novel perspective for relevant research. Many studies have examined the fascia and pain, and they have found that fascia stimulation only causes affective pain (such as pain, heaviness, and discomfort) [5] and that fascia chemical stimulation causes more pain and lasts longer than muscle stimulation [6]. These findings suggest that fascia is more linked to pain than muscle. Langevin et al. [34] found 20% less thoracolumbar fascia shear strain in chronic low back pain patients than in the normal population, suggesting a relationship between pain symptoms and fascia sliding capacity. The present study found that knee extensor eccentric exercise reduced vastus medialis and vastus lateralis deep fascial sliding capacity. However, Tenberg et al. [35] found that dumbbell elbow flexor eccentric exercise increased deep fascia thickness at 48, 72, and 96 h but did not affect fascia mobility. The contrast with the prior study may be attributable to exercise place and intensity. EIMD varies by exercise site, as established in earlier research [36]. This study employed 10 sets of 12 repetitions, twice the dose used by Tenberg et al. [35], who used 6 sets of 10 repetitions, which may have affected EIMD. This investigation found that TF reduced deep fascia slippage as expected. TF may cause “fascial shearing” in deep fascia sliding [17]. The theory claims that TF compression and active muscle contractions during movement induce mechanical stress and heat, which may transform the fascia from gel to fluid. Transition enhances fascia stretchability and mobility [18].

The mitigating effect of TF on the trend of increasing CK postexercise was observed in this study. CK, an indirect marker of muscle damage, is found in skeletal muscle and myocardium, but strenuous exercise damages cells and releases it into the blood, raising CK levels post-exercise [27]. TF may affect blood reperfusion to help CK levels recover. After ankle flossing 21 healthy students, Pasurka et al. [37] found a 30% increase in dorsal pedal artery blood perfusion. However, no significant changes were observed 60 minutes after exercise. In addition, Bohlen et al. [38] did not observe changes in blood flow following TF application. They conducted a 14-day TF intervention on the proximal and distal patella of the experimental leg in five participants and measured resting arterial inflow and reactive hyperemic blood flow after the 14-day intervention. Their results suggest that there may be only acute effects of TF on flow reperfusion. Thus, the acute changes in blood perfusion after TF may have reduced the level of CK.

Furthermore, this study supports earlier research [18, 39] indicating that the application of TF enhances strength, as evidenced by the comparison of knee extensor eccentric torque postexercise between the TF and CON groups. Eccentric torque was significantly greater in the TF group than in the CON group over time postexercise and returned to baseline level at 48 h postexercise. In a study by Kaneda et al. [18], participants were instructed to engage in active knee flexion, 20 times for three sets during the period of thigh wrapping. The maximal eccentric voluntary knee extension contraction of the intervention group (TF) was greater than that of the dynamic stretching group [18]. Konrad et al. [13] suggested that the enhancing effect of TF on performance may be due to its improvement in individual neuromuscular function. However, the specific mechanism still requires further investigation due to the lack of evidence.

This study provides scientific evidence supporting the application of TF to alleviate EIMD and DOMS after high-intensity exercise, which is a low-cost tool that can be recycled repeatedly. For exercisers, this intervention is simple and convenient, and is not restricted by space. To the best of our knowledge, this is the first study to explore the fascia shearing theory by observing the changes in deep fascia sliding before and after TF application. The results showed that high-intensity eccentric exercise decreased deep fascia sliding ability, but the TF effectively improved it.

The study’s limitations must be acknowledged. First, methodologically, TF’s placebo effect was not examined. Only male participants were recruited, so EIMD may affect females differently [40]. And in the present study, participants were physically inactive. Future research should examine how gender affects TF efficacy and its effects in physically active people. Due to high pressure, TF should be used with caution by cardiovascular disease patients. Second, this study measured deep fascia sliding pre- and post-exercise but not fascia thickness. Third, TF’s proper application is still discussed despite much research. The pressure range used in this study was based on earlier studies of joint RoM and performance, and different pressures of TF on the joint RoM and performance can cause different effects [13]. It is hypothesized that different pressures on TF affect the roles of EIMD and DOMS. Since active movement is required during compression, more research is needed to determine if TF’s effects are caused by compression, movement, or both.

## CONCLUSIONS

This study supports the effectiveness of TF as a recovery technique for alleviating EIMD. The current study shows that TF after high-intensity eccentric exercise reduces muscle damage and pain perception and improves eccentric strength recovery. Therefore, we recommend the use of TF for those who expect to reduce DOMS and promote strength recovery after exercise. The findings suggest that the protective effect of TF may be linked to enhancements in deep fascia sliding. Further studies are required to clarify the un derlying processes and to explore the effectiveness of TF in different populations and with various parameters.
